# Isolation of novel gut bifidobacteria using a combination of metagenomic and cultivation approaches

**DOI:** 10.1186/s13059-019-1711-6

**Published:** 2019-05-16

**Authors:** Gabriele Andrea Lugli, Christian Milani, Sabrina Duranti, Giulia Alessandri, Francesca Turroni, Leonardo Mancabelli, Danilo Tatoni, Maria Cristina Ossiprandi, Douwe van Sinderen, Marco Ventura

**Affiliations:** 1 Laboratory of Probiogenomics, Department of Chemistry, Life Sciences, and Environmental Sustainability, University of Parma, Parco Area delle Scienze 11a, 43124 Parma, Italy; 2 Department of Veterinary Medical Science, University of Parma, Parma, Italy; 3 Microbiome Research Hub, University of Parma, Parma, Italy; 4 APC Microbiome Institute and School of Microbiology, Bioscience Institute, National University of Ireland, Cork, Ireland

**Keywords:** Genomics, Metagenomics, Microbiota, Human gut commensals

## Abstract

Whole metagenome shotgun (WMGS) sequencing is a method that provides insights into the genomic composition and arrangement of complex microbial consortia. Here, we report how WMGS coupled with a cultivation approach allows the isolation of novel bifidobacteria from animal fecal samples. A combination of in silico analyses based on nucleotide and protein sequences facilitate the identification of genetic material belonging to putative novel species. Consequently, the prediction of metabolic properties by in silico analyses permits the identification of specific substrates that are then employed to isolate these species through a cultivation method.

**Electronic supplementary material:**

The online version of this article (10.1186/s13059-019-1711-6) contains supplementary material, which is available to authorized users.

## Background

Next-Generation Sequencing (NGS) technologies allow the generation of vast amounts of genomic data, facilitating a variety of DNA sequencing approaches that range from single genome sequencing to large-scale metagenomic studies [1]. While whole genome sequencing (WGS) reveals the complete genetic makeup of a specific organism, and the subsequent prediction of its biological features, the whole metagenome shotgun (WMGS) methodology provides genetic information about the abundant microorganisms present in a complex microbial consortium associated with a particular ecosystem based on the sequencing depth [2, 3]. Furthermore, through the reconstruction of sequenced DNA into consensus sequences, WMGS sequencing provides access to the genome content of yet uncultured bacteria, including novel species, which are otherwise very hard or even impossible to be identified by traditional culturing techniques [4–6].

Microorganisms are ubiquitous in nature, which means that they can be found everywhere. In this context, the human body, as well as that of non-human animals, is inhabited by a plethora of microbial species that may coexist with the host throughout its life span [7]. Most of the microbial communities that reside in the animal body are located in the large intestine, representing an estimated 10^14^ bacterial cells [8]. The gastrointestinal microbial community, also known as gut microbiota, exerts many important activities that support and preserve host health [9]. It is for this reason that the gut microbiota is the most extensively scrutinized microbial community (both in humans and other animals) through large-scale metagenomic studies [10]. As part of ongoing efforts to dissect the composition and associated activities of the gut microbiota, various studies have focused on the identification of novel bacterial species, whose genetic makeup is pivotal to unveil potential microbe-host interactions [11].

Recently, various strategies have been proposed for the enrichment of very low abundance strains from complex environmental matrices [12, 13]. However, these methodologies require a sequenced reference genome to perform DNA enrichment prior to sequencing. Besides, to explore such microbial dark matter, methodologies involving high-throughput culture conditions for the growth of bacteria followed by matrix-assisted laser desorption/ionization–time of flight (MALDI–TOF) or 16S rRNA amplification and sequencing have been employed [11, 14]. In this context, new bacterial species have been isolated, filling knowledge gaps regarding unknown microbial inhabitants of the human gut and allowing insights into the physiology of these taxa.

The focus of the current study was to apply WMGS sequencing in order to investigate the presence of novel gut commensals species belonging to the genus *Bifidobacterium* among the gut microbiota of animals. For this purpose, we sequenced and analyzed samples collected from banteng (*Bos javanicus*), Goeldi’s marmoset (*Callimico goeldii*), and pygmy marmoset (*Callithrix pygmaea*) due to the high abundance of putative novel species of the genus *Bifidobacterium* as based on a previous study [15]. We therefore employed a custom-made METAnnotatorX pipeline [16] to screen the sequencing data of each sample in order to retrieve genomic dark matter that was predicted to belong to the genus *Bifidobacterium*.

## Results and discussion

**Fig. 1:**
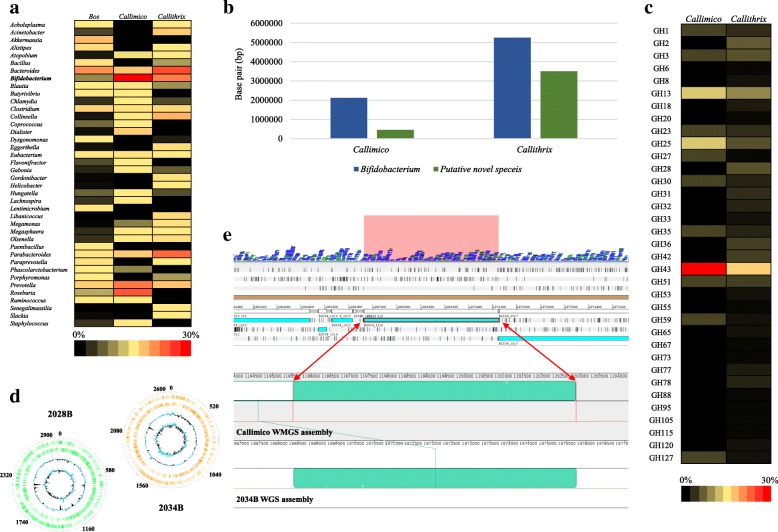
Identification of novel bacterial strains belonging to the genus *Bifidobacterium*. **a** The relative abundance of the reconstructed bacterial genomic material at genus level obtained from *Bos javanicus*, *Callimico goeldii*, and *Callithrix pygmaea* samples. Only genera that display at least 0.2% of the total amount of the assembled data were included in the heat map. **b** The abundance of putative novel genetic material belonging to the genus *Bifidobacterium* retrieved by means of the custom METAnnotatorX pipeline. The *y*-axis shows the number of base pairs (bp) assigned to the genus *Bifidobacterium*. Total assembled bifidobacterial genome sequences are reported in blue, while putative novel bifidobacterial sequences are highlighted in green. **c** The relative abundance of GH enzymes predicted from the unclassified bifidobacterial genetic material retrieved from *Callimico* and *Callithrix* WMGS sequencing. **d** A circular genome atlas of *Bifidobacterium* 2028B and 2034B. External circles denote gene positions within genomes, while internal circles describe G + C% deviation and GC skew (G-C/G + C). **e** A genomic region of *Bifidobacterium* 2034B in which the gene encoding a pullulanase was identified, a predicted property that was subsequently used for cultivation-based glycan selection. The sequence coverage of the data obtained from WMGS sequencing is reported in the top margin, while in the bottom margin the alignment with the reconstructed genomes obtained between WMGS and WGS sequencing is indicated

WMGS sequencing of animal stool samples produced approximately 79 million of paired-end reads with an average length of ~ 150 bp (see Additional file 1: Supplementary Materials and Additional file 2: Table S1), which were analyzed through the METAnnotatorX pipeline. A preliminary screening of the obtained sequence reads revealed marked variations in the relative abundance of bifidobacteria between different samples analyzed, ranging from 0.1% in the *Bos* sample to 22.3 and 25% for *Callithrix* and *Callimico* samples, respectively (Fig. 1a). Due to the low abundance of bifidobacterial reads in the *Bos* sample, the metagenomic data were used to perform a validation screening aimed at revealing the minimum amount of genomic DNA needed to detect a specific taxon (Additional file 3: Figure S1). In the case of *Callithrix* and *Callimico*, metagenomic data were assembled, revealing more than 800 contigs (with a length of > 5000 bp) predicted to belong to the genus *Bifidobacterium*, taxonomically classified by means of the proteome of each contig (Fig. 1b).

To identify genomic contigs that putatively belong to unclassified bifidobacterial taxa, a custom script employing the results of the METAnnotatorX pipeline was implemented (Additional file 3: Figure S2). Starting from the collected bifidobacterial contigs, a comparison against three databases based on each bifidobacterial genomic sequence was performed (see Additional file 1: Supplementary Materials). Gene homology/protein similarity searches at both nucleotide and deduced protein level were carried out coupled with chromosomal sequence comparisons to discard contigs attributed to known species and closely related taxa. Thus, collected contigs belonging to unknown bifidobacterial species were reduced to 435 by manual removal of phage and plasmid sequences (Fig. 1b).

Predicted genes among the selected contigs were compared to a Glycosyl Hydrolase (GH) database to assess the glycobiome of the putative unknown bifidobacterial species. Based on the thus generated glycobiomes (Additional file 2: Table S2), we predicted that four glycans, i.e., arabinogalactan, pullulan, starch, and xylan, represented carbon sources for these putative novel bifidobacterial species (Fig. 1c). Thus, various cultivation experiments were performed, where aliquots of fecal samples from *Callimico* and *Callithrix* were added to a chemically defined medium (CDM), containing a specific glycan, as indicated above, as its sole carbon source (see Additional file 1: Supplementary Materials). These carbohydrate-specific cultivation experiments allowed the growth of 13 phenotypically different bifidobacterial isolates, which were able to metabolize the selected glycans. Subsequently, amplification and sequencing of the internal transcribed spacer (ITS) sequence of these isolates was performed, and the obtained ITS sequences were compared to a previously described ITS bifidobacterial database [15] (Additional file 2: Table S3). This procedure allowed the identification of two strains that do not belong to previously characterized bifidobacterial species [17]. The latter putative novel bifidobacterial isolates, named 2028B and 2034B, were subjected to WGS, which generated two genomes with a size of 2.96 and 2.61 Mb, respectively (Fig. 1d and Additional file 2: Table S4). Accordingly, novel bifidobacterial strains 2028B (=LMG 30938= CCUG 72814) and 2034B (=LMG 30939= CCUG 72815) were submitted to two public culture collections [18]. The reconstruction of these genomes highlighted the presence of specific genes predicted to be responsible for the metabolism of the employed carbohydrate substrates as identified in the WMGS analyses, such as pullulanases and beta xylosidases. To validate the proposed approach, additional experiments were performed based on selective enrichment with inclusion in the medium of glucose, ribose, xylan, and pullulan as its unique carbon source based on the identified genes mentioned above (see Additional file 1: Supplementary Materials and Additional file 3: Figure S3). We observed more rigorous growth of strains 2028B and 2034B when cultivated on complex carbon sources, such as xylan and pullulan, as compared to glucose (Additional file 3: Figure S3a, S3b and S3c). Furthermore, the addition of complex carbon sources, i.e., xylan and pullulan, directly into the *Callimico* fecal sample resulted in an enrichment of these two strains, in particular strain 2034B in combination with pullulan, resulting in a one log increase in bacterial abundance as compared to medium containing glucose (i.e., from 8 × 10^5^ to 4 × 10^6^) (Additional file 3: Figure S3d). Despite the observed specificity in the isolation procedure of the two novel strains, it is worth mentioning that further microorganisms may grow in the selective media. To avoid this issue, mupirocin was added to the CDM (see Additional file 1: Supplementary Materials).

The average nucleotide identity (ANI) analysis of the here decoded genomes with all so far known bifidobacterial (sub)species [19], highlighted that strain 2028B possesses a 92.29% ANI value with respect to *Bifidobacterium vansinderenii* LMG 30126, while isolate 2034B exhibits an 87.32% ANI value with respect to *Bifidobacterium biavatii* DSM 23969 (Additional file 2: Table S5). Notably, two bacterial strains displaying an ANI value < 95% are considered to belong to distinct species [20]. Mapping WMGS reads among the reconstructed genome sequences of strains 2028B and 2034B revealed that both genomes were entirely covered by the sequenced paired-end reads of the *Callimico* sample with an average coverage of 8.8 and 8, respectively. Furthermore, alignment of the reconstructed chromosomes of strains 2028B and 2034B with the deduced contigs belonging to unknown bifidobacterial species of the *Callimico* sample allowed the identification of contigs that belong to the novel assembled genomes (Fig. 1e). Accordingly, the genetic repertoire of strains 2028B and 2034B, coupled to their metabolic abilities, allowed the isolation of these novel *Bifidobacterium* taxa.

**Fig. 2:**
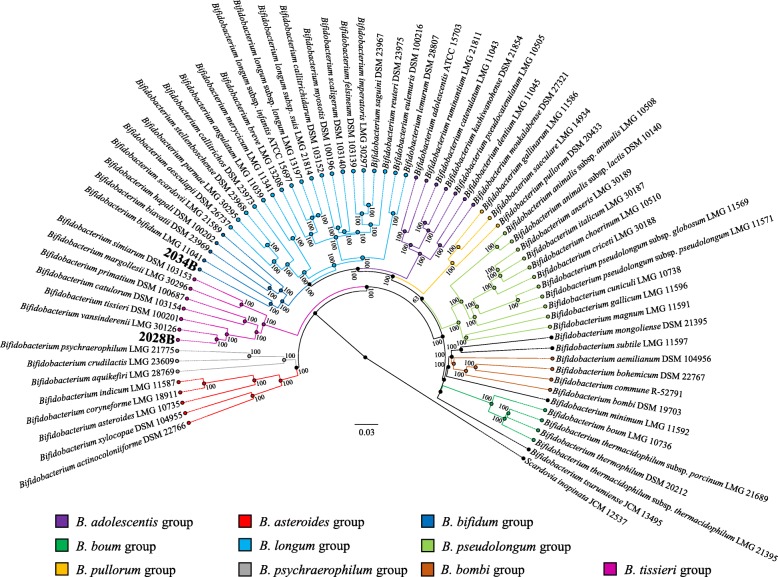
Phylogenomic tree of the genus *Bifidobacterium* based on the concatenation of 233 core gene (and derived protein) sequences from genomes of novel isolates 2028B and 2034B, and 69 type strains of the genus *Bifidobacterium*. The amino acid-deduced core gene-based tree highlights the division into 10 phylogenetic groups represented by different colors. The phylogenetic tree was constructed by the neighbor-joining method, with the genome sequence of *Scardovia inopinata* JCM 12537 as outgroup. Bootstrap percentages above 50 are shown at node points, based on 1000 replicates of the phylogenetic tree

The availability of 2028B and 2034B genome sequences also allowed us to investigate their phylogenetic relationship with each of the 69 currently recognized bifidobacterial taxa [19, 21]. A comparative genome analysis was undertaken to highlight orthologous genes between sequenced type strains of the genus *Bifidobacterium*, resulting in 31,520 clusters of orthologous genes (COGs). The analyses allowed us to identify 261 COGs that were shared among all genomes, representing the bifidobacterial core genome. The concatenation of 233 core gene protein sequences (excluding 28 paralogs that were identified among type strains) allowed the construction of a bifidobacterial phylogenetic tree (Fig. 2). As shown in Fig. 2, strain 2034B clustered in the *Bifidobacterium bifidum* phylogenetic group [19], which also contains *B. biavatii* DSM 23969, whose relatedness has been highlighted in the ANI analysis (see above). Besides, strain 2028B grouped together with *B. vansinderenii* LMG 30126. Thus, based on these phylogenomic analyses, the relatedness among bifidobacterial type strains allowed the identification of a new phylogenetic cluster, which consists of strain 2028B plus six strains isolated from various monkey species [19, 21–23], here proposed to constitute the *Bifidobacterium tissieri* group (Fig. 2).

## Conclusions

In the current study, we demonstrated how the implementation of selected tools for the identification of putative novel bacterial taxa from WMGS sequencing data allowed insights into the microbial dark matter of the mammalian gut. Based on the scientific field of interest, this approach can be applied to any bacterial genus for which several genome sequences have been decoded and for which there is just minimal knowledge on associated nutritional requirements. Thus, the predicted genetic makeup informs cultivation attempts to facilitate isolation of novel species of the examined genus. This approach was successfully applied to unravel the dark matter concerning key mammalian gut commensals belonging to the genus *Bifidobacterium* [15], ultimately resulting in the identification of two novel bifidobacterial species.

## Additional files

Additional file 1: Supplementary materials. (DOCX 56 kb)

Additional file 2: **Table S1.** Metagenomic sequence data. **Table S2.** Predicted glycobiome of novel species. **Table S3.** ITS sequences of the 15 bifidobacterial isolates. **Table S4.** General genetic features. **Table S5.** Average Nucleotide Identity between bifidobacterial taxa. (XLSX 78 kb)

Additional file 3: **Figure S1.** Evaluation of the accuracy of METAnnotatorX in the detection of a specific species mixed within WMGS sequencing data of *Bos javanicus*. **Figure S2.** Workflow of the experiments. **Figure S3.** Viable cell counts and enrichment of novel bifidobacterial strains based on different carbon sources. **Figure S4.** Validation of the custom METAnnotatorX pipeline for the identification of novel bacterial species. (DOCX 1306 kb)

## Abbreviations

ANI: Average nucleotide identity
CDM: Chemically defined medium
GH: Glycosyl hydrolases
ITS: Internal transcribed spacer
MRS: de Man-Rogosa-Sharpe
NGS: Next-generation sequencing
WGS: Whole genome sequencing
WMGS: Whole metagenome shotgun
