# Supplementary material for: Isolation of novel gut bifidobacteria using a combination of metagenomic and cultivation approaches
Source: Genome Biol. 2019 May 16;20:96. doi: 10.1186/s13059-019-1711-6 (PMC6524291; doi:10.1186/s13059-019-1711-6)
Supplement: Supplementary file 1 — Supplementary materials. (DOCX 56 kb) [file 13059_2019_1711_MOESM1_ESM.docx]

**Supplementary Materials**

**Microbial genome sequences.** More than 500 bifidobacterial genome sequences were retrieved from the National Center for Biotechnology Information (NCBI) public database. These genomic sequences were used as input for the MEGAnnotator pipeline [[1](#_ENREF_1)] in order to predict protein-encoding open reading frames (ORFs) using the same methodology and parameters for each genome.

**DNA extraction and ITS sequencing.** Fecal samples were subjected to DNA extraction using the QIAmp DNA Stool Mini Kit following the manufacturer’s instructions (Qiagen). DNA concentration and purity were then determined employing a Picodrop microtitre Spectrophotometer (Picodrop).

The Internal Transcribe Spacer (ITS) sequences were amplified from extracted DNA using the specific primer pair ProbioBif-ITS_Fw (5’-CCTTGTACACACCGCCCG-3’) and ProbioBif-ITS_Rev (5’-AGATGTTTCACTTCCCTGCG-3’), which targets the variable region between the 16S rRNA and 23S rRNA gene sequences. Each PCR mixture (25 μl) contained 1.5 mM of MgCl_2_, 20 mM of Tris-HCl, 50 mM of KCl, 200 μM of each deoxynucleoside triphosphate, 25 pmol of each of the two primers, 1 U of Taq DNA polymerase (Taq PCR master mix kit; Biotechrabbit GmbH, Germany), and 50 ng of DNA template. Each PCR cycling program consisted of an initial denaturation step of 5 min at 95°C, followed by amplification for 30 cycles as follows: denaturation (30 s at 95°C), annealing (1 min at 60°C), and extension (1 min at 72°C). The PCR was completed with a single elongation step (5 min at 72°C). The resulting amplicons were separated on 1 % agarose gels, followed by SYBR safe staining. PCR amplicons were purified using the QIAquick PCR Purification kit (Qiagen, United Kingdom) and subsequently sequenced. Nucleotide sequencing of ITS region was performed by Eurofins Mix2Seq Kits service (Eurofins Genomics, Germany) using 16S_bif-SEQ1 (5’-CGTCAAGTCATGAAAGTGGG-3’).

**Shotgun metagenomics.** DNA library preparation was performed using the Nextera XT DNA sample preparation kit (Illumina, San Diego, CA) according to the manufacturer's instructions. One ng input DNA from each sample was used for library preparation. The isolated DNA underwent fragmentation, adapter ligation and amplification. The ready-to-go libraries were pooled equimolarly, denaturated and diluted to a sequencing concentration of 1.5 pM. Sequencing was performed on NextSeq 550 instrument (Illumina, San Diego, CA), according to the manufacturer's instructions, using the 2x150 bp High Output sequencing kit, and spike-in of 1 % PhiX control library.

**Metagenomic analyses.** To improve data set quality of each individual sample, a ﬁltering step was performed to obtain only high-quality reads (minimum mean quality score 20, window size 5, quality threshold 25 and minimum length 80) using the fastq-mcf script (https://github.com/ExpressionAnalysis/ea-utils/blob/wiki/FastqMcf.md). Collected filtered reads were assembled using SPAdes v3.12 [[2](#_ENREF_2)] and the resulting contigs were taxonomically classified based on homology searches deﬁned by means of RAPSearch2 (Reduced Alphabet based Protein similarity Search 2) [[3](#_ENREF_3)] based on RefSeq NCBI databases. The above-mentioned steps, as well as the taxonomic classification of the filtered paired-end reads were independently performed by the METAnnotatorX pipeline [[4](#_ENREF_4)].

**Bifidobacterial genome reconstruction.** Paired fastq files of shotgun metagenomics, as well as those of WGS sequencing of bifidobacterial strains were used as input for SPAdes assembler v3.12 [[2](#_ENREF_2)], for *de novo* metagenomic assemblies using default parameters enabling the metagenomic flag option (-meta). Based on the length of the paired-end reads, SPAdes parameters were coupled with minimum k-mer sizes of 21, 33, and 55 (100 bp reads length) to a maximum of 77, 99 and 127 (150-250 bp read length). ORFs of each assembled contig were predicted with Prodigal [[5](#_ENREF_5)] and annotated by means of the software MEGAnnotator [[1](#_ENREF_1)].

**Implementation of a custom METAnnotatorX pipeline.** To identify genetic material of putative novel species of the genus *Bifidobacterium* from WMGS sequencing data, a script was implemented to discard assembled data derived from known species and closely related taxa. Primarily, the script requires as input taxonomically classified reconstructed contigs that were generated by METAnnotatorX at genus level [[4](#_ENREF_4)]. Subsequent to this, a peptide database composed of all deduced coding sequences of the *Bifidobacterium* genus was employed to taxonomically classify each predicted gene (stringent cut-offs were applied: e-value cutoff of 1e^-30^ and minimum length of the alignment 30 amino acids). Gene homology searches were deﬁned by means of RAPSearch2, while default parameters of METAnnotatorX were used to assign the bacterial taxonomy at species level of each contig. Those contigs that were classified as known bifidobacterial species were discarded from the analysis. Then, a second database was built with chromosomal sequences of the retrieved bifidobacterial strains from the NCBI database. By means of BLAST algorithm, we selected those contigs that displayed nucleotide identity values below 94 % with respect to sequenced bifidobacterial strains. Finally, a third database was built based on nucleotide sequences of the genus *Bifidobacterium*, to perform homology searches employing predicted genes of putative novel contigs of the genus. BLAST algorithm was employed once more and genes that showed amino acid identity values below 90 % were collected. In this manner we obtained a pool of contigs composed of genomic sequences that display the highest variability in terms of sequence identity to known taxa of this genus. In this custom version of the METAnnotatorX script, the identity value cutoff used in the chromosomal and gene analyses was introduced as variables that could easily be modified starting the script. The custom script can be downloaded from the Probiogenomics lab website (http://probiogenomics.unipr.it/pbi).

**Validation of the methodology.** The pipeline was validated using a public dataset (NCBI BioProject ID: PRJNA63661) composed by fecal samples of infants that were previously recognized to be rich in bifidobacterial content [[6](#_ENREF_6), [7](#_ENREF_7)]. The complete METAnnotatorX pipeline was used to taxonomically classify the assembled bifidobacterial contigs at species level, resulting in a high prevalence of contigs classified as *Bifidobacterium longum* (Additional file 3: Fig. S4). Thus, we developed nucleotide and protein databases employing all bifidobacterial sequenced strains except for *B. longum* sequences. Using this approach, we were able to apply our pipeline treating *B. longum* as an unknown species of the genus. Results showed that the total number of the assembled contigs taxonomically classified as *B. longum*, by means of the METAnnotatorX pipeline, ends in the pool of unknown contigs of the genus *Bifidobacterium* with our custom script (Additional file 3: Fig. S4). Based on these results, we expected that novel species of the genus *Bifidobacterium* can be retrieved in a similar fashion from a WMGS sequencing analysis of biological samples.

**Evaluation of the minimum amount of metagenomic data needed to perform a robust metagenomic assembly.** Metagenomic data of *Bos* sample were used to identify the number of reads necessary to obtain a valuable assembly of a specific bacterial species. To pursue this goal, 14 pools of 30 million reads were assembled with a variable distribution of *Bifidobacterium choerinum* paired-end reads, ranging from 10,000 (0.03 % of the total amount of reads) to 6,000,000 (20 %) paired-end reads. The choice of the bifidobacterial strain used for this screening was based on its absence within the metagenomic *Bos* sample. Thus, the METAnnotatorX pipeline was employed to perform read and contig classification of the 14 read pools. Profiling of *B. choerium* at read level showed a similar number of reads when compared to the hypothetical distribution based on the number of reads inserted in each pool (Additional file 3: Fig. S1). Interestingly, assembled contigs belonging to *B. choerium* were reported at 60,000 reads corresponding to 0.2 % of the total amount of paired-end reads of the *Bos* sample (Fig. 1). Afterward, the genome length of the reconstructed *B. choerium* reaches a plateau starting from 300,000 reads corresponding to 1 % of the total amount of paired-end reads of the *Bos* sample. Thus, we assume that the above reported values correspond to the minimum number of reads (150 bp paired-end reads) needed to identify a bacterial contig and the minimum amount of reads for a proper genome assembly.

**Genomic *in silico* analyses.** The reconstructed metagenomic contigs were screened to unveil genes encoding glycoside hydrolase (GH) enzymes by means of the CAZy database [[8](#_ENREF_8)]. Filtered metagenomic reads were used to identify the coverage of the novel bifidobacterial taxa by means of Bowtie 2 [[9](#_ENREF_9)] through multiple-hit mapping and “very-sensitive” policy. The mapping was performed using a minimum score threshold function (–score-min C,-13,0) in order to limit reads of arbitrary length to two mismatches and retain those matches with at least 98 % full-length identity. Bifidobacterial average genome coverage has been calculated by means of bbmap software (<https://github.com/BioInfoTools/BBMap>), mapping metagenomic sequenced reads with 99 % sequence identity (minid=0.99). Genome sequence alignments were performed using MAUVE software [[10](#_ENREF_10)]. Average Nucleotide Identity (ANI) values were calculated using the program JSpecies version 1.2.1 [[11](#_ENREF_11)] between genome sequences of the two novel bifidobacterial isolates and all currently known type strains of the genus *Bifidobacterium* (Additional file 2: Table S5).

**Comparative genomics.** A pan-genome calculation was performed using the pan-genome analysis pipeline PGAP [[12](#_ENREF_12)], including 69 genomes of corresponding bifidobacterial type strains collected from the NCBI database and genomes of the two novel strains isolated in this study. Each predicted proteome of a given bifidobacterial strain was screened for orthologues against the proteome of every collected genome by means of BLAST analysis [[13](#_ENREF_13)] (cutoff: E value of 1×10^−5^ and 50 % identity over at least 80 % of both protein sequences). The resulting output was then clustered into protein families by means of MCL (graph theory-based Markov clustering algorithm) [[14](#_ENREF_14)], using the gene family (GF) method. Protein families shared between analyzed genomes allowed us to identify the core genome of the *Bifidobacterium* genus. Each set of orthologous proteins, belonging to the core genome, was aligned using Mafft software [[15](#_ENREF_15)], and a phylogenetic tree was constructed using ClustalW [[16](#_ENREF_16)]. Based on this comparative genomic analysis, a bifidobacterial supertree was constructed and visualized using FigTree (<http://tree.bio.ed.ac.uk/software/figtree/>) (Fig. 2).

**Bifidobacterial isolation.** One gram of a *Bos javanicus*, *Callimico goeldii* or *Callithrix pygmaea* fecal sample was mixed with nine ml of phosphate-buffered saline (PBS), pH 6.5. Serial dilutions and subsequent platings were performed using four different selective media. Each medium consists of a chemically defined medium (CDM) with the addition of 50 μg/ml mupirocin (Delchimica, Italy), 0.05 % (wt/col) L-cysteine hydrochloride and one specific carbohydrate such as arabinogalactan, or pullulan, or starch or xylan. CDM contains (per liter of distilled water) 4.0 g of sodium acetate; 1.0 g of tri-ammonium citrate; 2.0 g of KH_2_PO_4_; 2.0 g of K_2_HPO_4_; 0.5 g of MgSO_4_.7H_2_O; 0.05 g of MnSO_4_.H_2_O; 0.02 g of FeSO_4_.7H_2_O; 0.2 g of CaCl_2_; 20 mg of adenine; 40 mg of xanthine; 0.4 g of cysteine; 0.3 g of aspartic acid; 0.3 g of glutamic acid; 0.2 g of each the following amino acids: alanine, arginine, glycine, histidine, isoleucine, leucine, lysine, methionine, phenylalanine, proline, serine, threonine, tryptophan, tyrosine, and valine; 0.5 g of orotic acid; 0.5 mg of *p*-aminobenzoic acid; 0.5 mg of folic acid, 2.0 mg of nicotinic acid; 2.0 mg of Ca-pantothenate; 1.0 mg of biotin; 2.0 mg of pyridoxal; 2.0 mg of riboflavin; and 1.0 mg of vitamin B_12_. The medium was sterilized by filtration (0.22 μm). Agar plates were incubated for 48 h at 37°C in a chamber (Concept 400; Ruskin) with an anaerobic atmosphere (2.99 % H_2_, 17.01 % CO_2_ and 80 % N_2_). Morphologically distinct colonies that developed on CDM plates were randomly picked and re-streaked to isolate purified bacterial strains. All isolates were subjected to DNA isolation and characterized as described in the DNA extraction and ITS sequencing section.

**Fermentation profiles.** Novel identified bifidobacterial taxa were grown on specific carbon sources. In particular, de Man-Rogosa-Sharpe (MRS) medium without glucose was supplemented with 1 % (wt/vol) of a specific sugar as mentioned above. Cultures were cultivated in the wells of a 96-well microtiter plate, with each well containing a different sugar, and incubated in an anaerobic cabinet at 37°C. The optical densities at 600nm (OD600) was determined using a plate reader (Biotek, VT, USA) at two different time points, 24 and 48 h. Growth assays were carried out in duplicate and non-inoculated MRS medium was used as a negative control. Carbohydrates tested in this study include arabinogalactan, glucose, pullulan, ribose, starch xylose and xylan (Additional file 3: Fig. S3a). Furthermore, total cell counts of 2028B and 2034B strain growth on CDM supplemented with glucose, ribose, xylan or pullulan as a sole carbon source (Additional file 3: Fig. S3b and Fig. S3c), at 48 hours after the inoculum, were evaluated by viable cell count.

**Evaluation of the novel bifidobacterial cell density by qPCR.** qRT-PCR reactions were performed on MicroAmp optical plates sealed with MicroAmp optical caps (Applied Biosystems, Foster City, 2 CA) and amplifications were carried out on a CFX96 system (BioRad, CA, USA). Primers used in this study are 2028Bpk_Fw (5’-GTTCCGTAGTGCGCTGAGTC-3’) and 2028Bpk_Rv (5’-TATCGACGTTCCATTCGTCA-3’) based on a putative protein kinase-coding gene of the 2028B strain, and 2034Bap_Fw (5’-ACCGCAGATACTGTCGATCC-3’) and 2034Bap_Rv (5’-ACCGTACCGTTGGAGTTGAG-3’) based on a predicted amylopullulanase-encoding gene of the 2034B strain. Both primer pairs were designed on unique gene sequences retrieved from the comparative genomic analysis performed among the 69 bifidobacterial type strain genomes collected from the NCBI database. Each qPCR assay was performed as previously described [[17](#_ENREF_17)]. A standard curve was generated using CFX96 software (BioRad). Chromosomal DNA extracted from 2028B and 2034B cultures was used for standard curves. All experiments were executed in triplicate in at least two independent PCR runs. In each run, negative controls (water) for each primer set were included. qPCR was performed on aliquots of fecal sample from *Callimico* growth on CDM supplemented with glucose, ribose, xylan or pullulan as a sole carbon source (Additional file 3: Fig. S3d).

**Evaluation of the cell density of the novel bifidobacteria by flow cytometry assay.** Total cell counts of 2028B and 2034B cultures grown on CDM supplemented with glucose, ribose, xylan or pullulan as a sole carbon source (Additional file 3: Fig. S3b and Fig. S3c), at 48 hours after the inoculum, were evaluated by flow cytometry with an Attune^TM^ NxT (ThermoFisher Scientific) equipped with a Blue Laser set at 50 mWatt and tuned to an excitation wavelength of 488 nm. Bacterial cells were stained with 1 μL/mL SYBR^®^ Green I (1:100 dilution in DMSO; Molecular Probes), and incubated in the dark for at least 15 min before measurement. Multiparametric analyses were performed on both scattering signals (FSC, SSC) and SYBR Green I fluorescence was detected on FL1 channel. Cell debris was excluded from acquisition analysis by a sample-specific FL1 threshold. All data were statistically analyzed with Attune NxT Flow Cytometer Software.

**Supplementary reference**

1. Lugli GA, Milani C, Mancabelli L, van Sinderen D, Ventura M: **MEGAnnotator: a user-friendly pipeline for microbial genomes assembly and annotation.** *FEMS Microbiol Lett* 2016, **363**.

2. Bankevich A, Nurk S, Antipov D, Gurevich AA, Dvorkin M, Kulikov AS, Lesin VM, Nikolenko SI, Pham S, Prjibelski AD, et al: **SPAdes: a new genome assembly algorithm and its applications to single-cell sequencing.** *J Comput Biol* 2012, **19:**455-477.

3. Zhao Y, Tang H, Ye Y: **RAPSearch2: a fast and memory-efficient protein similarity search tool for next-generation sequencing data.** *Bioinformatics* 2012, **28:**125-126.

4. Milani C, Casey E, Lugli GA, Moore R, Kaczorowska J, Feehily C, Mangifesta M, Mancabelli L, Duranti S, Turroni F, et al: **Tracing mother-infant transmission of bacteriophages by means of a novel analytical tool for shotgun metagenomic datasets: METAnnotatorX.** *Microbiome* 2018, **6:**145.

5. Hyatt D, Chen GL, Locascio PF, Land ML, Larimer FW, Hauser LJ: **Prodigal: prokaryotic gene recognition and translation initiation site identification.** *BMC Bioinformatics* 2010, **11:**119.

6. Lugli GA, Milani C, Turroni F, Tremblay D, Ferrario C, Mancabelli L, Duranti S, Ward DV, Ossiprandi MC, Moineau S, et al: **Prophages of the genus Bifidobacterium as modulating agents of the infant gut microbiota.** *Environ Microbiol* 2016, **18:**2196-2213.

7. Duranti S, Lugli GA, Mancabelli L, Turroni F, Milani C, Mangifesta M, Ferrario C, Anzalone R, Viappiani A, van Sinderen D, Ventura M: **Prevalence of Antibiotic Resistance Genes among Human Gut-Derived Bifidobacteria.** *Appl Environ Microbiol* 2017, **83**.

8. Lombard V, Golaconda Ramulu H, Drula E, Coutinho PM, Henrissat B: **The carbohydrate-active enzymes database (CAZy) in 2013.** *Nucleic Acids Res* 2014, **42:**D490-495.

9. Langmead B, Salzberg SL: **Fast gapped-read alignment with Bowtie 2.** *Nat Methods* 2012, **9:**357-359.

10. Darling AE, Mau B, Perna NT: **progressiveMauve: multiple genome alignment with gene gain, loss and rearrangement.** *PLoS One* 2010, **5:**e11147.

11. Richter M, Rossello-Mora R, Oliver Glockner F, Peplies J: **JSpeciesWS: a web server for prokaryotic species circumscription based on pairwise genome comparison.** *Bioinformatics* 2016, **32:**929-931.

12. Zhao Y, Wu J, Yang J, Sun S, Xiao J, Yu J: **PGAP: pan-genomes analysis pipeline.** *Bioinformatics* 2012, **28:**416-418.

13. Altschul SF, Gish W, Miller W, Myers EW, Lipman DJ: **Basic local alignment search tool.** *J Mol Biol* 1990, **215:**403-410.

14. Enright AJ, Van Dongen S, Ouzounis CA: **An efficient algorithm for large-scale detection of protein families.** *Nucleic Acids Res* 2002, **30:**1575-1584.

15. Katoh K, Misawa K, Kuma K, Miyata T: **MAFFT: a novel method for rapid multiple sequence alignment based on fast Fourier transform.** *Nucleic Acids Res* 2002, **30:**3059-3066.

16. Larkin MA, Blackshields G, Brown NP, Chenna R, McGettigan PA, McWilliam H, Valentin F, Wallace IM, Wilm A, Lopez R, et al: **Clustal W and Clustal X version 2.0.** *Bioinformatics* 2007, **23:**2947-2948.

17. Milani C, Mangifesta M, Mancabelli L, Lugli GA, James K, Duranti S, Turroni F, Ferrario C, Ossiprandi MC, van Sinderen D, Ventura M: **Unveiling bifidobacterial biogeography across the mammalian branch of the tree of life.** *ISME J* 2017.
