# Supplementary material for: Isolation of novel gut bifidobacteria using a combination of metagenomic and cultivation approaches
Source: Genome Biol. 2019 May 16;20:96. doi: 10.1186/s13059-019-1711-6 (PMC6524291; doi:10.1186/s13059-019-1711-6)
Supplement: Supplementary file 3 — Figure S1. Evaluation of the accuracy of METAnnotatorX in the detection of a specific species mixed within WMGS sequencing data of Bos javanicus. Figure S2. Workflow of the experiments. Figure S3. Viable cell counts and enrichment of novel bifidobacterial strains based on different carbon sources. Figure S4. Validation of the custom METAnnotatorX pipeline for the identification of novel bacterial species. (DOCX 1306 kb) [file 13059_2019_1711_MOESM3_ESM.docx]

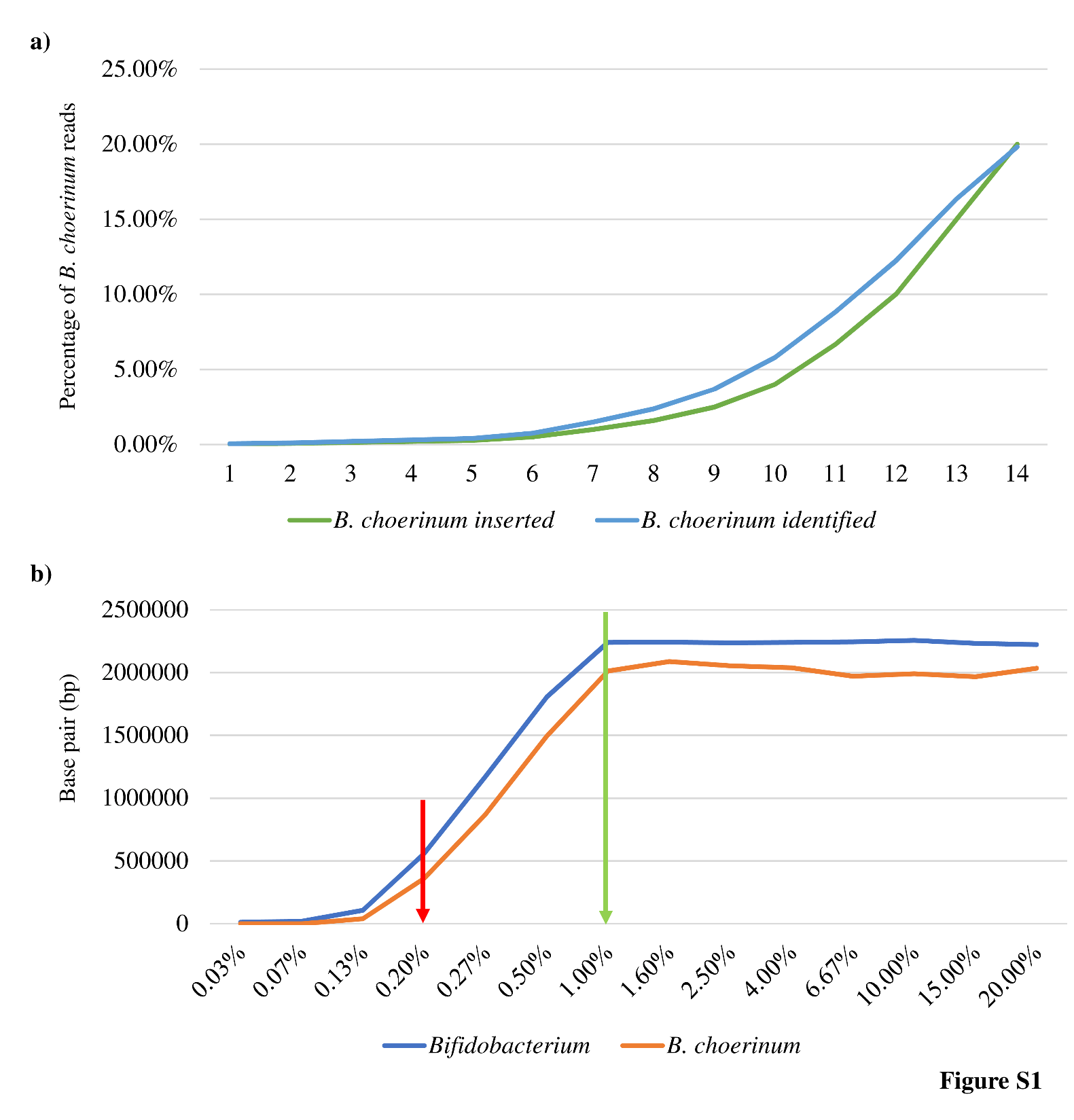


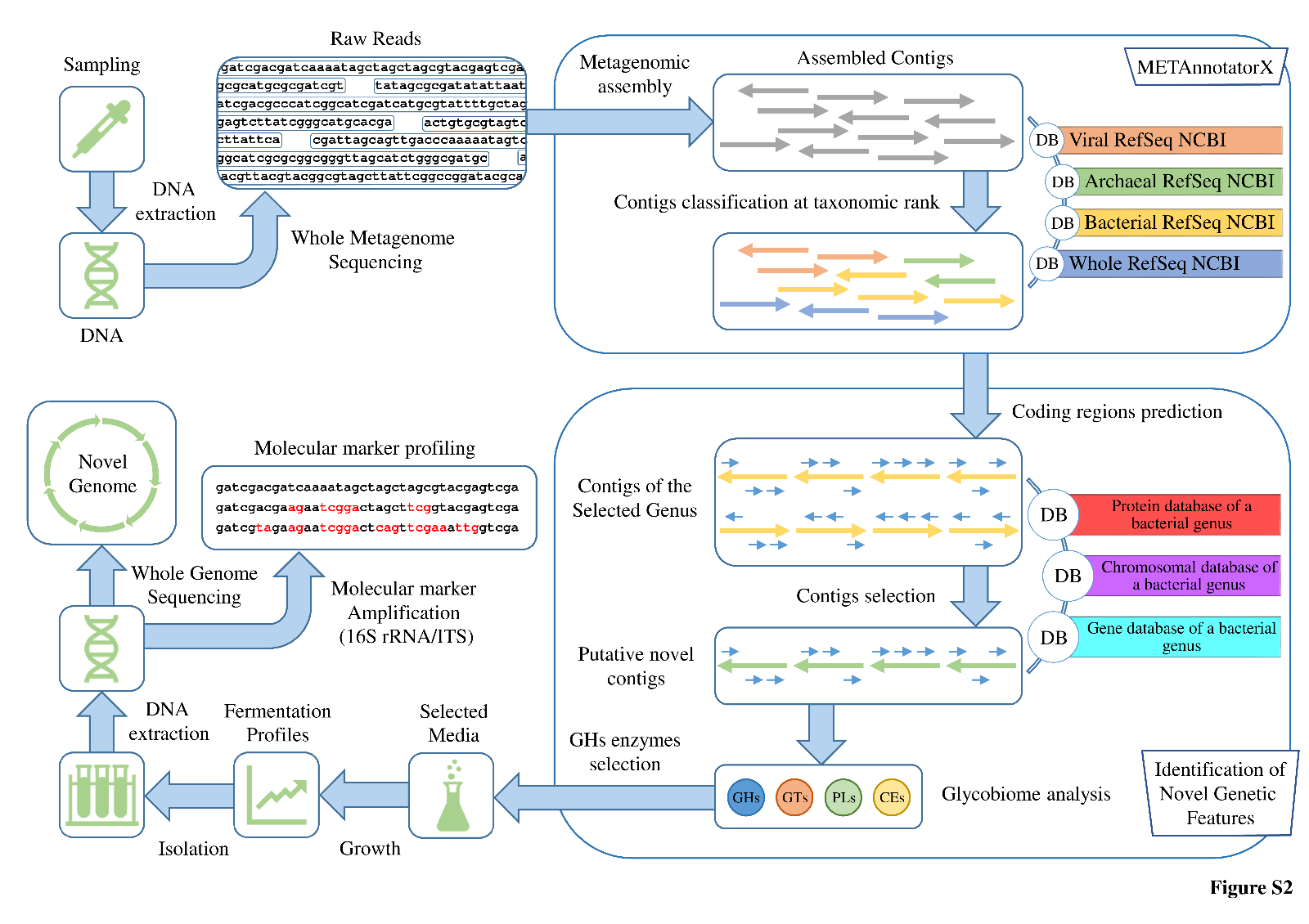


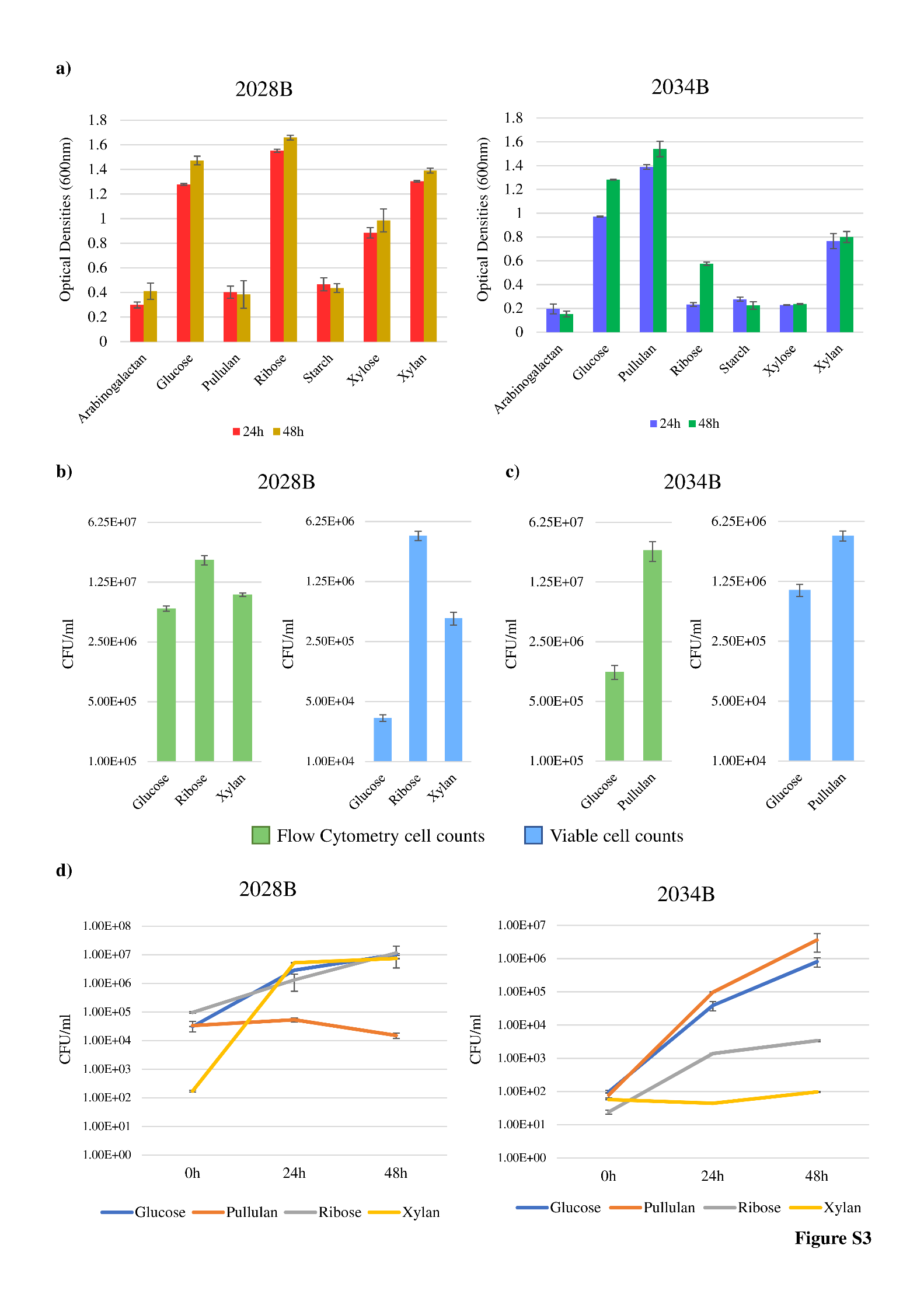


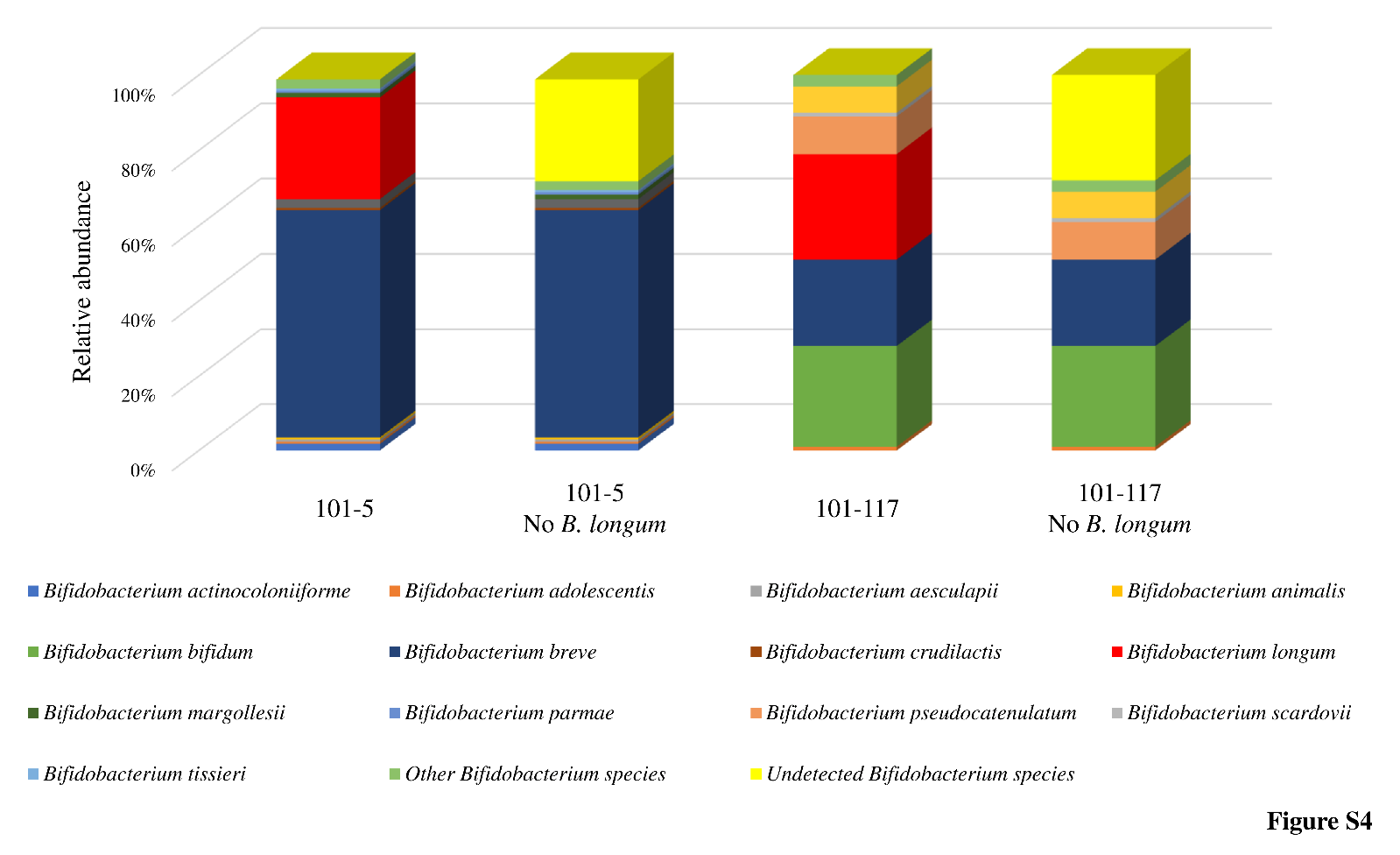


**Figure S1.** Evaluation of the accuracy of METAnnotatorX in the detection of a specific species mixed within WMGS sequencing data of *Bos javanicus*. Panel a shows the expected (green) and observed (blue) abundancies of *B. choerinum* within 14 sub-sets of the *Bos javanicus* metagenomic data. Genetic material belonging to *B. choerinum* was added incrementally as reported on the *y-axis*. Panel b displays the reconstructed genome portions of bifidobacterial species (blue) and those of *B. choerinum* (orange) within 14 sub-sets of the *Bos javanicus* metagenomic data. Genetic material belonging to *B. choerinum* was added incrementally as reported on the *y-axis*. The minimum number of paired-end reads needed to detect *B. choerinum* is indicated with a red arrow, while the minimum number required to obtain its complete genome is indicated with a green arrow.

**Figure S2.** Workflow of the experiments. Starting from biological samples, whole metagenomics shotgun sequencing was performed on extracted DNA obtaining a pool of paired-end reads. The METAnnotatorX pipeline was further employed for metagenomic data assembly and taxonomic classification of the resulting contigs at genus level. Thus, gene prediction was performed on a pool of contigs that are predicted to belong to a specific bacterial genus. A combination of databases built on nucleotide and protein sequences of the selected bacterial genus allowed us to identify those contigs that belong to putative novel species. Consequently, carbohydrate substrates for growth of bifidobacterial were selected based on a glycobiome analysis of the putative novel species, allowing the identification of those glycoside hydrolase enzymes predicted within contigs. Based on the fermentation profiles obtained, bacterial strains were isolated and further analyzed by the sequencing of a molecular marker. Finally, those strains displaying a unique profile were subjected to whole genome sequencing in order to facilitate genomic classification of the novel bacterial taxa.

**Figure S3.** Viable cell counts and enrichment of novel bifidobacterial strains based on different carbon sources. Panel a shows the fermentation profiles of 2028B and 2034B based on de Man-Rogosa-Sharpe (MRS) medium without glucose supplemented with either arabinogalactan, glucose, pullulan, ribose, starch, xylose or xylan at 24 and 48h. The optical densities at 600nm (OD600) in the y-axis represent the means of three independent experiments conducted in duplicate. The vertical bars indicate standard deviations. Panel b exhibits growth of strain 2028B after 48 h based on CDM supplemented with glucose, ribose or xylan as unique carbon source. Flow cytometry counts are highlighted in green, while viable cell counts are represented in blue. The cell count number in the y-axis represents the means of three independent experiments reported in logarithmic scale. Panel c depicts the same charts for strain 2034B. Panel d displays the cell density of strains 2028B and 2034B determined by qRT-PCR on aliquots of fecal sample from *Callimico* growth on CDM supplemented with glucose, ribose, xylan and pullulan as unique carbon source. The number of viable cells in the y-axis represent the means of three independent experiments reported in logarithmic scale.

**Figure S4.** Validation of the custom METAnnotatorX pipeline for the identification of novel bacterial species. The proﬁles of the *Bifidobacterium* population obtained from two infant fecal samples named 101-5 and 101-117 (NCBI BioProject ID: PRJNA63661) are depicted in the corresponding bar plot. The relative abundance of each bifidobacterial species is based on the length of the reconstructed contigs of the reported species respect to the total amount of assembled DNA of the genus *Bifidobacterium*. Pillars with the tag “No *B. longum*” were analyzed removing *B. longum* strains from the database. Species below 1 % are collapsed in “Other *Bifidobacterium* species”.
